# Developmental expression and evolution of hexamerin and haemocyanin from *Folsomia candida* (Collembola)

**DOI:** 10.1111/imb.12585

**Published:** 2019-05-08

**Authors:** Y. Liang, W. Xie, Y.‐X. Luan

**Affiliations:** ^1^ Key Laboratory of Insect Developmental and Evolutionary Biology Shanghai Institute of Plant Physiology and Ecology, Chinese Academy of Sciences Shanghai China; ^2^ School of Biological and Chemical Sciences, Queen Mary University of London London UK; ^3^ Guangdong Provincial Key Laboratory of Insect Developmental Biology and Applied Technology Institute of Insect Science and Technology, School of Life Sciences, South China Normal University Guangzhou China

**Keywords:** Collembola, hexamerin, haemocyanin, developmental transcriptomes, molecular phylogeny, molecular clock

## Abstract

Haemocyanins constitute a group of copper‐containing respiratory proteins, and hexamerins were derived from hexapod haemocyanin but lost the ability to transport oxygen and serve as storage proteins. Although hexamerins have been reported in most insect species, none of them has been identified in Collembola, one of the most primitive hexapod lineages, thereby preventing us from exploring relevant evolutionary scenarios regarding the origin and evolution of hexamerins in hexapods. Here we report on collembolan hexamerins for the first time, and investigated the temporal expression profiles of hexamerin and haemocyanin in the collembolan *Folsomia candida*. Haemocyanin was expressed over the entire life cycle, with higher expression at the embryonic stage than at other stages, whereas hexamerin expression was restricted to embryos, unlike insect hexamerins, which are generally expressed from larval to adult stages. A phylogenetic analysis and molecular clock estimation suggested that all investigated hexapod hexamerins have a single and ancient origin (~423 Ma), coincident with the rise of atmospheric oxygen levels in the Silurian–Devonian period, indicating a physiological link between molecular evolution and Palaeozoic oxygen changes.

## Introduction

The arthropod haemocyanin superfamily includes haemocyanins, phenoloxidases, pseudohaemocyanins/cryptocyanins, hexamerins and hexamerin receptors, which share similar structures and a common ancestor (Burmester, 2002). Haemocyanins are respiratory proteins that can transport O_2_ and float freely in the haemolymph of chelicerates (Rehm *et al.*, 2012), myriapods (Kusche and Burmester, 2001), crustaceans (Malacostraca, Remipedia, Ostracoda and Branchiura) (Ertas *et al.*, 2009; Marxen *et al.*, 2014; Pinnow *et al.*, 2016), collembolans (Flachsbarth *et al.*, 2017) and some primitive insect orders (Pick *et al.*, 2009). Hexamerins and cryptocyanins are storage proteins originating from haemocyanins that specifically occur in hexapods and malacostracan crustaceans, respectively. In hexamerins and cryptocyanins, the six common histidine sites for Cu^2+^ binding are mutated, and these proteins can no longer serve as oxygen transporters (Burmester, 2002). The functions of hexamerins are mostly associated with moulting cycles or nutritional conditions (Telfer and Kunkel, 1991; Burmester, 2001; Chandrasekar *et al*., 2009). Specifically, hexamerins are synthesized in the fat body and then released into the haemolymph at high levels during the nonfeeding periods of insects to support their development (Manohar *et al.*, 2010).

Although numerous hexamerins have been reported in various hemi‐/holometabolous insects (Beintema *et al.*, 1994; Capurro *et al.*, 2000), few hexamerins from ametabolous species have been identified to date (Pick and Burmester, 2009; Xie and Luan, 2014), particularly in basal hexapods, which is indispensable for exploring the origin and evolution of hexamerins. As one of the earliest hexapod groups, Collembola (springtails) is one of the most widespread and abundant terrestrial arthropod taxa and plays a key role in understanding the origin of insects (Hopkin, 1997; Nardi *et al.*, 2003; Carapelli *et al.*, 2006).

Here, we report the occurrence of hexamerins in Collembola for the first time, reveal the developmental expression profiles of haemocyanin and hexamerin from the collembolan *Folsomia candida* and further identify their co‐expressed genes to speculate on their putative functions by time‐wise transcriptome analysis. In addition, by incorporating the new data, we have conducted a comprehensive phylogenetic reconstruction and molecular evolutionary analyses of hexamerins, haemocyanins and cryptocyanins in the scope of Pancrustacea. The possible evolutionary driving force for the occurrence of hexamerins in hexapods is also discussed. Our results provide crucial hints regarding the origin of hexamerins and their functional adaptation in hexapods.

## Results

### Identification of putative hexamerins and haemocyanins from collembolans and diplurans

We explored haemocyanin and hexamerin sequences in basal hexapods (Collembola, Diplura and Protura) by PCR cloning, rapid amplification of cDNA ends (RACE) and mining from transcriptomes/genomes (our unpublished data; Misof *et al.*, 2014; Faddeeva *et al.*, 2015). In total, we report 17 new sequences here (with nine complete sequences shown in bold in Table 1): three collembolan hexamerins, 10 dipluran hexamerins and four collembolan haemocyanins (Table 1). Amongst these sequences, two collembolan hexamerins [Fca(DK)Hx1 from *F. candida* (Denmark strain, DK; hexamerin, Hx) and Fca(SH)Hx1 from *F. candida* (Shanghai strain, SH)] and eight dipluran hexamerins (LweHx2, LweHx3, LweHx4 and LweHx5 from *Lepidocampa weberi*; OjaHx4 from *Occasjapyx japonicus*; OsiHx1, OsiHx2 and OsiHx3 from *Octostigma sinensis*) are totally new, whereas the others from Collembola and Diplura were previously predicted as haemocyanins, haemocyanin‐like or hexamerin‐like in published transcriptomes (Misof *et al.*, 2014; Faddeeva *et al.*, 2015). In this study, we validated and re‐annotated these sequences as haemocyanin 1 (Hc1), haemocyanin 2 (Hc2) and hexamerin (Hx), respectively, according to the results of phylogenetic analysis (Table 1).

**Table 1:** Properties of 17 putative hexamerin (Hx) and haemocyanin (Hc) molecules newly reported or annotated in this study (with nine complete sequences shown in bold)

Species			Sequence name	Deduced amino acid length	GenBank accession no.	Source and method
Collembola	Isotomidae	*Folsomia candida* (Denmark, strain)	Fca(DK)Hx1	**694**	MG930994	blast analysis of our transcriptomes (unpublished), PCR & RACE
		*Folsomia candida* (Shanghai, strain)	Fca(SH)Hx1	**700**	MG930995	
	Entomobryidae	*Orchesella cincta*	OciHx1	517	ODN00731.1 (HcA chain)	blast analysis of a transcriptome from Faddeeva *et al. *(2015)
			OciHc1	494	ODN01444.1 (HcE chain)	
			OciHc2	**677**	ODN00732.1 (HcB chain)	
	Tomoceridae	*Pogonognathellus* sp. AD‐2013	PloHc1	**678**	MG931002	blast analysis of transcriptomes from Misof *et al. *(2014)
			PloHc2	**678**	MG931003	
Diplura	Campodeidae	*Campodea augens*	CauHx1	469	MG930992	blast analysis of transcriptomes from Misof *et al. *(2014)
			CauHx2	397	MG930993	
		*Lepidocampa weberi*	LweHx2	**690**	MG930996	blast analysis of our transcriptomes (unpublished), PCR & RACE
			LweHx3	**681**	MG930997	
			LweHx4	601	MG930998	
			LweHx5	570	MG930990	
	Japygidae	*Occasjapyx japonicus*	OjaHx4	421	MG930991	PCR& RACE
	Octostigmatide	*Octostigma sinensis*	OsiHx1	**680**	MG930999	blast analysis of our transcriptomes (unpublished), PCR & RACE
			OsiHx2	419	MG931000	
			OsiHx3	**681**	MG931001	

RACE, rapid amplification of cDNA ends.

**Figure 1:**
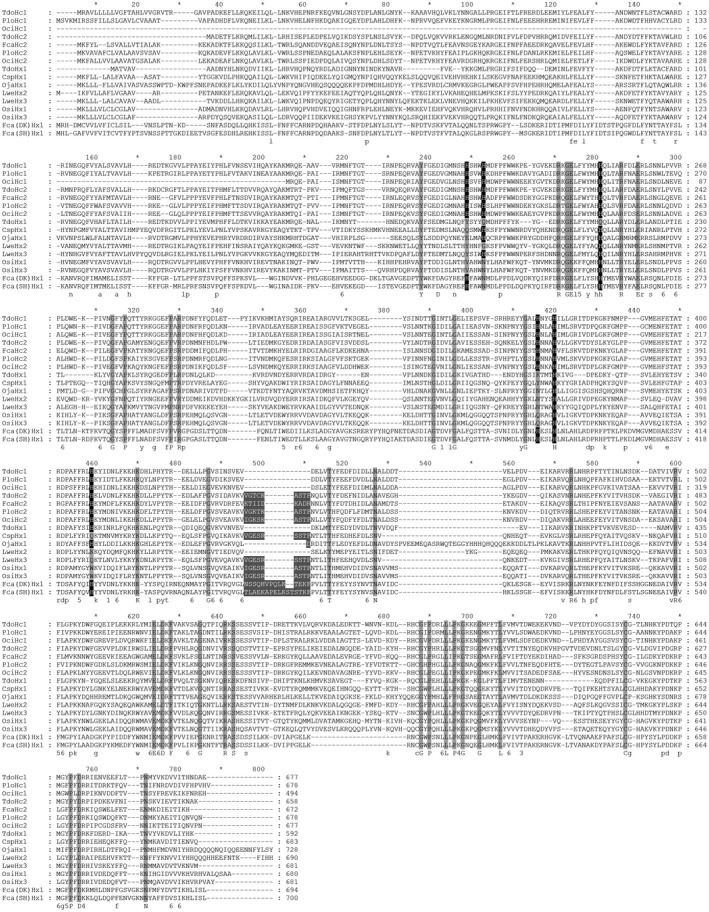
Multiple sequence alignment of hexamerins and haemocyanins. Six new hexamerins from the collembolans *Folsomia candida* (Denmark strain) [Fca(DK)Hx1] and *F. candida* (Shanghai strain) [Fca(SH)Hx1] and the diplurans *Octostigma sinensis* (OsiHx1 and OsiHx3) and *Lepidocampa weberi* (LweHx2 and LweHx3) and four new haemocyanins from the collembolans *Orchesella cincta* (OciHc1 and OciHc2) and *Pogonognathellus* sp. AD‐2013 (PloHc1 and PloHc2) are compared with three previously reported hexamerins from the zygentoman *Thermobia domestica* (TdoHx; Pick *et al.*, 2008) and the diplurans *Campodea* sp. (CspHex1; Pick and Burmester, 2009) and *Occasjapyx japonicus* (OjaHx1; Xie and Luan, 2014), as well as three known haemocyanins from the zygentoman *T. domestica* (TdoHc1 and TdoHc2; Pick *et al.*, 2008) and the collembolan *F. candida* (FcaHc2; Xie and Luan, 2014). All sequences were aligned with mafft (Katoh *et al.*, 2017) and visualized using genedoc (v. 2.7; Nicholas *et al*., 1997). The six conserved histidine sites for Cu^2+^ binding in haemocyanins are shaded in black; an insertion of nine amino acids is shaded in dark grey; and other strictly conserved residues are shaded in light grey.

For the first time, we cloned two complete cDNA sequences of putative collembolan hexamerins, Fca(DK)Hx1 and Fca(SH)Hx1, which contain 694 and 700 amino acids, respectively, and have both lost the second histidine of the corresponding six conserved histidine residues for O_2 _binding in haemocyanins (Fig. 1, shaded grey in the black columns). We did not find any other hexamerins from these two strains of *F*. *candida* despite comprehensively screening their transcriptomes and genomes (Misof *et al.*, 2014; Faddeeva *et al.*, 2015; our unpublished data) and conducting PCR amplification of samples at different developmental stages.

Hexamerins have been reported from two dipluran groups: Campodeoidea and Japygoidea (Xie and Luan, 2014). Here, we obtained three new hexamerins (OsiHx1, OsiHx2 and OsiHx3) from *Oct. sinensis*, representing the third dipluran group: Projapygoidea. Thus, all three groups of Diplura have hexamerins. Unlike collembolans, most dipluran species contain more than one hexamerin (Tables 1 and S3), and their hexamerins lost more than one histidine in the corresponding residues for O_2 _binding in haemocyanins (Fig. 1). We failed to find any haemocyanins in diplurans.

Hc1 has been reported in *F*. *candida* (Pick *et al.*, 2009), but we found only the Hc2 sequence in *F*. *candida* (DK), which is consistent with the results reported by Flachsbarth *et al. *(2017). In addition, we did not obtain any Hc1 or Hc2 sequences from *F*. *candida* (SH). These results may be because of the use of different strains of *F*. *candida*, as the species is widely distributed around the world with obvious genetic variation.

The insertion of nine amino acids in Hc2, which was thought to represent an obvious difference between hexapod Hc1 and Hc2 (Ertas *et al.*, 2009), is also conserved in the collembolan Hc1 and Hc2 sequences reported in this study (alignment positions 496–511, highlighted in dark grey in Fig. 1). Most previously reported hexamerins do not contain this insertion, except for several dipluran hexamerins (Pick and Burmester, 2009; Xie and Luan, 2014). We further observed insertions at the same alignment positions of the newly discovered collembolan and dipluran hexamerins, consisting of 14, 16, nine, nine and nine amino acids in Fca(DK)Hx1, Fca(SH)Hx1, LweHx3, OsiHx1 and OsiHx3, respectively, whereas no such insertion was observed in LweHx2. This suggests that a specific insertion could have occurred in the common ancestor of hexapod Hc1 and Hc2 but was lost later in the ancestor of hexapod Hc1. However, it is also possible that these insertions in hexapod Hc2 and basal hexapod hexamerins evolved independently as the insertion sequences present in collembolan and dipluran hexamerins are not conserved.

### Developmental expression profiles of the putative hexamerin and haemocyanin in the collembolan *F. candida*

As we found both hexamerin and haemocyanin in the collembolan *F. candida* (DK), we wanted to elucidate the expression profiles of *Fca(DK)Hx1* and *Fca(DK)Hc2* during development. 31 samples of *F. candida* (DK) covering all developmental stages from the first to the 31st day (a life cycle, including eggs, juveniles and adults) were collected for semi‐quantitative reverse transcription PCR (RT‐PCR). In addition, 15 samples covering eggs (each day from the days 0.5 to 9.5), juveniles (days 12.5, 19.5 and 28.5) and adults (days 31.5 and 45.5) were collected for transcriptome sequencing and measurement of transcript abundance (reads per kilobase per million, RPKM). Information about the transcriptome data obtained is presented in Table 2 (datasets can be downloaded from the National Center for Biotechnology Information (NCBI), Short Read Archive accession ID: SRP132624; BioProject: PRJNA433725).

**Table 2:** Summary of developmental transcriptome sequencing and assemblies for *Folsomia candida* (Denmark strain).

Sample	Number of raw reads	Number of clean reads	Mapped/clean reads (%)	SRA number
Egg‐0.5d	24 136 075	24 123 643	97.17	SRR6705488
Egg‐1.5d	24 135 617	24 121 361	97.50	SRR6705489
Egg‐2.5d	24 136 452	24 120 477	95.76	SRR6705486
Egg‐3.5d	24 136 211	24 123 527	97.01	SRR6705487
Egg‐4.5d	24 136 074	24 124 256	97.03	SRR6705484
Egg‐5.5d	24 136 222	24 122 509	96.94	SRR6705485
Egg‐6.5d	24 136 442	24 124 834	96.33	SRR6705482
Egg‐7.5d	24 136 521	24 119 154	96.57	SRR6705483
Egg‐8.5d	24 136 376	24 124 146	96.66	SRR6705480
Egg‐9.5d	24 136 194	24 125 505	96.68	SRR6705481
Juv‐12.5d	24 136 024	24 122 314	96.39	SRR6705478
Juv‐19.5d	24 136 552	24 119 643	96.29	SRR6705479
Juv‐28.5d	24 136 529	24 115 524	96.25	SRR6705476
Adu‐31.5d	24 136 497	24 122 489	96.60	SRR6705477
Adu‐45.5d	24 136 728	24 126 584	97.22	SRR6705475

SRA, Short Read Archive; d, days after egg laying; Juv, juvenile; Adu, adult.

**Figure 2:**
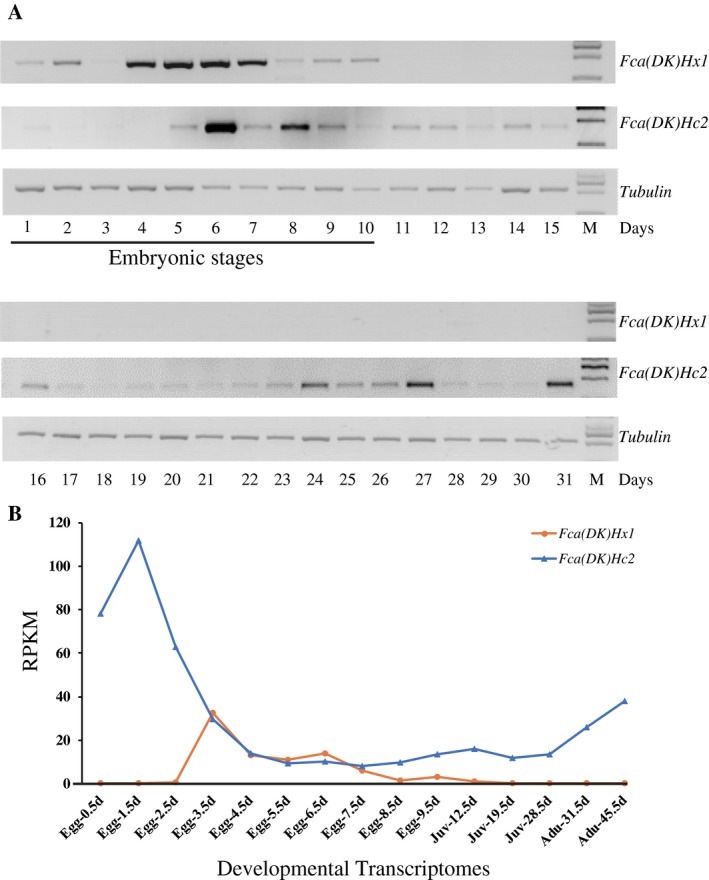
Expression profiles of *Folsomia candida (Denmark strain*) *hexamerin 1 *[*Fca(DK)Hx1*]/* Fca(DK) haemocyanin 2* [*Fca(DK)Hc2*] messenger RNA (mRNA) during the life cycle of *F. candida* (DK). (A) Analysis of gene expression patterns for 31 days after egg laying were determined via reverse transcription PCR; samples included embryos (first to 10th days), juveniles (11th to 30th days) and adults (31st day). *Tubulin* was used as the control gene to normalize RNA abundance. (B) Quantification of normalized mRNA expression from the transcriptomes of embryos (days 0.5–9.5), juveniles (days 12.5, 19.5 and 28.5) and adults (days 31.5 and 45.5). Reads per kilobase per million (RPKM) values were used to normalize the raw count by transcript length and sequencing depth.

Expression profiles of collembolan *Fca(DK)Hc2* and *Fca(DK)Hx1* were found to be consistent between the RT‐PCR (Fig. 2A) and transcriptome analyses (Fig. 2B). Interestingly, the expression of *Fca(DK)Hx1* was limited to the embryonic stages. Specifically, the expression level increased after 0.5 days, reached a peak at the middle stage of embryonic development and decreased slowly thereafter; no expression was detectable after hatching. A similar embryonic‐specific expression pattern was confirmed for *Fca(SH)Hx1* based on the transcriptome analysis from different developmental stages of *F. candida* (SH) (unpublished data from our laboratory). Accordingly, our results indicate that collembolan hexamerins are embryo*‐*specific proteins, unlike most insect hexamerins, which are primarily expressed during the larval and adult stages (Wolfe *et al.*, 1977; Manohar *et al.*, 2010; Martins *et al.*, 2010, 2011; Burmester, 2015b; Martins and Bitondi, 2016). *Fca(DK)Hc2* was detected during all embryonic, juvenile and adult stages, but had the highest expression during the embryonic stage. The haemocyanins of the migratory locust also have high expression in embryos (Chen *et al.*, 2015), indicating that the role of haemocyanin in embryonic development is probably conserved at least in some hexapods.

### Gene co‐expression analyses of the putative hexamerin and haemocyanin in the collembolan *F. candida*

Constructing a co‐expression network using large‐scale gene expression data is an effective way to uncover new biological knowledge (Kumari *et al.*, 2012). In order to identify genes co‐expressed with putative collembolan hexamerin and haemocyanin, we used the Spearman correlation method to mine genes correlated with *Fca(DK)Hx1* and *Fca(DK)Hc2*, respectively, via developmental transcriptome data from 10 embryonic stages.

There were 127 genes co‐expressed (*r* ^2^ ≥ 0.9, *P* ≤ 0.05) with *Fca(DK)Hx1* (Dataset S1), and their gene ontology (GO) molecular functions were annotated as oxidoreductase activity, ion transmembrane transporter activity, ion binding, transaminase activity, hydrolase activity and nucleotide binding (Fig. S1). Notably, *methyl farnesoate epoxidase* was highly correlated with *Fca(DK)Hx1* (*r* ^2^ = 0.91, *P* ≤ 0.01), and was annotated in the biological processes of juvenile hormone (JH) biosynthesis (Fig. S1). A correlation between hexamerin and JH has also been confirmed in the termite *Reticulitermes flavipes* (Zhou *et al.*, 2007). In addition, *acid phosphatase type 7 *was also highly correlated with *Fca(DK)Hx1* (*r* ^2^ = 0.94, *P* ≤ 0.01), and Kyoto encyclopedia of genes and genomes (KEGG) pathway annotated it in the riboflavin metabolism pathway (Table S1). As previously reported, riboflavin‐binding hexamerins are present at high concentrations in the haemolymph of moth pupae (Magee *et al.*, 1994; Pan and Telfer, 1999). Although gene co‐expression is not *bona fide* evidence for functions, our analyses may give some indications for future functional studies.

There were 1069 genes co‐expressed (*r* ^2^ ≥ 0.9, *P* ≤ 0.05; Dataset S2) with *Fca(DK)Hc2.* So many co‐expressed genes may indicate that *Fca(DK)Hc2* is indispensable as a respiratory protein. However, there were few intersections between the genes co‐expressed with* Fca(DK)Hx1* and those with *Fca(DK)Hc2* (Fig. S2 and Table S2), suggesting that they play distinct roles during the development of *F*. *candida*.

### Strong purifying selection on hexapod hexamerins and haemocyanins

The ratio of nonsynonymous (*d*_N_) to synonymous (*d*_S_) substitution rates (*ω* = *d*_N_/*d*_S_) reflects natural selection at the molecular level, with *ω* = 1, *ω* < 1 and *ω* > 1 indicating neutral evolution, purifying selection and positive selection, respectively (Yang and Nielsen, 2002), which could reflect the evolutionary conservation and functional constraint of a gene. By using a one‐ratio model (M0 model, one* ω* for all sites in all branches; Goldman and Yang, 1994), we found the *ω* values for hexapod Hc and Hx were significantly less than 1: 0.012 for Hc1, 0.003 for Hc2 and 0.104 for Hx, demonstrating that both hexapod Hc and Hx have evolved under strong purifying selection, and suggesting that they were very conserved during the evolution of hexapods. In addition, hexapod Hc2 is observed to be under stronger purifying selection than Hc1, indicating that Hc2 plays a more essential role than Hc1 as the conserved respiratory protein, and the relatively relaxed evolutionary constraint on Hc1 facilitated the origin of hexamerins.

### Phylogenetic analysis and molecular dating of hexapod hexamerins

**Figure 3:**
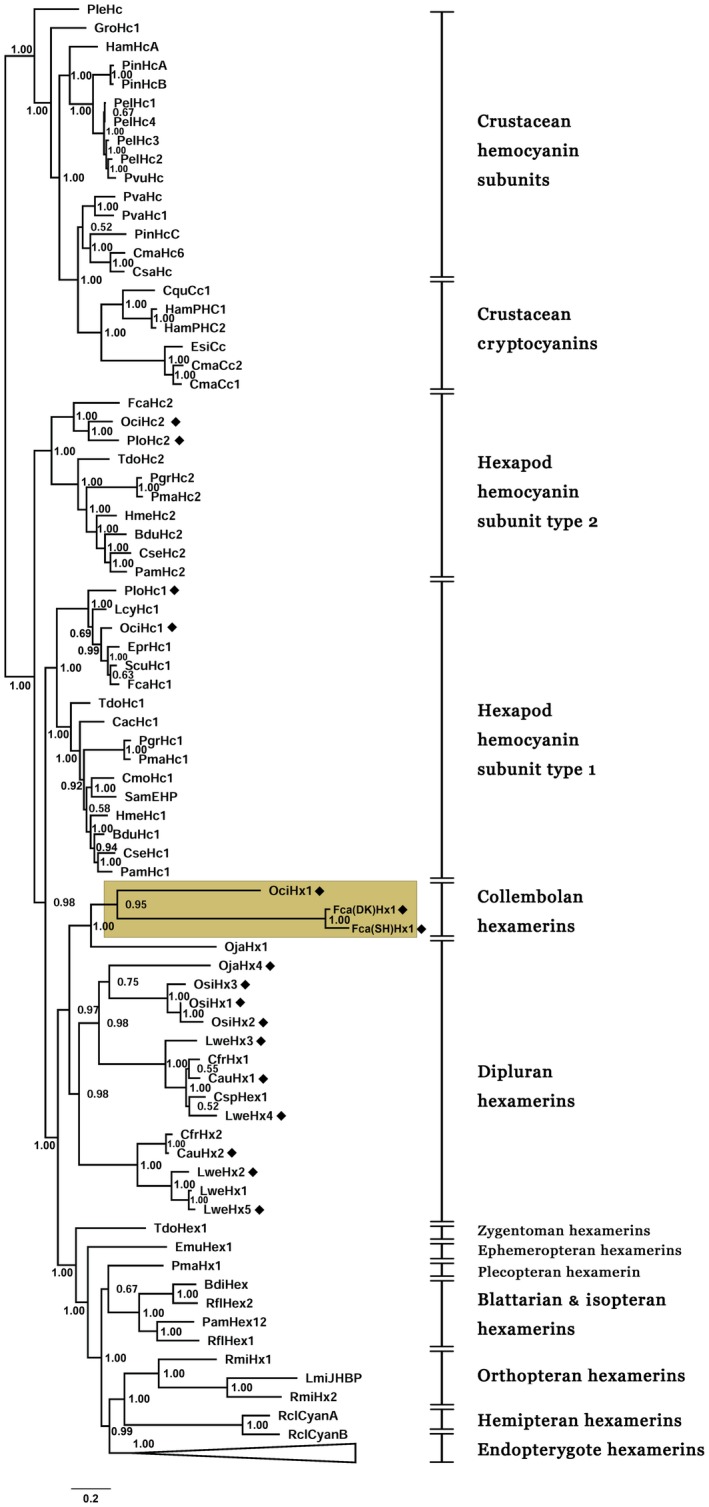
Bayesian analysis of pancrustacean haemocyanins and storage proteins. Crustacean molecules were regarded as the outgroup, and the tree was calculated using mrbayes at the Cyberinfrastructure for Phylogenetic Research (CIPRES) Science Gateway. The phylogenetic tree was based on 130 protein sequences, including 17 new sequences provided in Table 1, 109 sequences from Xie and Luan (2014) and four cryptocyanins from Terwilliger *et al.* (1999), Terwilliger and Ryan (2006), Wang X. and Feng Z. (unpublished data, GenBank accession no. AGH32536) and Glazer *et al.* (2015). The clades of endopterygote hexamerins were collapsed to simplify the topology. For the original tree please refer to Fig. S3. The scale bar denotes 0.2 amino acid substitutions per site. Numbers at the nodes indicate the Bayesian posterior probabilities. New sequences added in this study are marked with black squares. The full species names and protein names for all abbreviations are listed in Tables 1 and S3.

We constructed an alignment for phylogenetic analysis of haemocyanin family members in pancrustaceans, including 113 previously reported sequences (Table S3) and 17 new sequences reported in this study (Table 1 and Dataset S3). In our phylogenetic tree reconstructed by Bayesian analysis (Figs 3 and S3), the collembolan hexamerins clustered with dipluran hexamerins, and they formed a sister clade to all insect hexamerins. Accordingly, a single origin of hexamerins in Hexapoda is further supported. Four new collembolan haemocyanins were included in the clades of hexapod haemocyanin subunits type 1 and 2, respectively (Fig. 3). Therefore, the phylogenetic inferences suggested that both gene duplication events (Hc1 and Hc2, Hc1 and Hx) occurred before the split of Collembola and other hexapods. The topology of the phylogenetic tree reconstructed with the maximum likelihood method was exactly the same, although the support values were relatively lower (data not shown).

**Figure 4:**
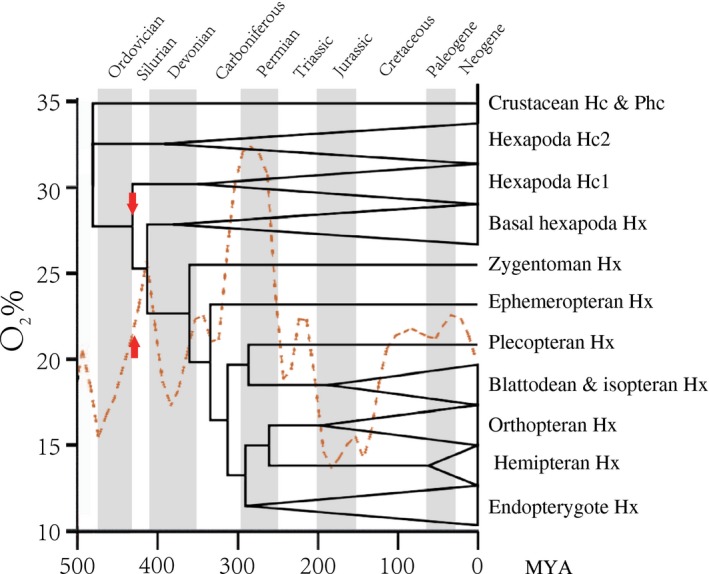
Molecular dating of hexamerins. A simplified phylogenetic topology was derived from the phylogenetic tree constructed with the maximum likelihood method. The names of geological periods are labelled at the top (Burmester, 2015a)*.* The red arrows suggest the divergence time of hexapod hexamerins at approximately 423 Ma. The dashed curve shows the estimated Phanerozoic atmospheric O_2 _concentration (Berner, 2009). Hc, haemocyanin; Hx, hexamerin; Phc, pseudohaemocyanin.

With relevant fossil calibration points applied, we performed a molecular clock analysis using the reconstructed phylogenetic tree. Our molecular dating results suggested that haemocyanin subunit type 1 and type 2 split approximately 473 Ma, and the hexapod hexamerins evolved from haemocyanins approximately 423 Ma (Fig. 4). According to the molecular divergence of hexamerins, the basal hexapods emerged approximately 411 Ma; the stem group of insects arose approximately 359 Ma; and the winged insects first occurred approximately 333 Ma. The estimated dates were similar to those calculated from Pick *et al.* (2008) and the fossil record (Whalley and Jarzembowski, 1981). When the Palaeozoic oxygen concentration curve (Berner, 2009) was anchored to the time‐calibrated phylogenetic tree (Fig. 4), we observed that the origin of hexapod hexamerins coincided with increasing oxygen concentration during the late Silurian–early Devonian period.

## Discussion

### Occurrence of haemocyanins and hexamerins in basal hexapods

Although most insects have developed an advanced tracheal system, many of them still need respiratory proteins (haemocyanin or haemoglobin; Burmester, 2015a) to transport additional oxygen to produce energy. However, amongst basal hexapod groups, no respiratory protein has yet been detected in Protura and Diplura, despite our efforts based on molecular cloning and transcriptome analysis. Although most proturan species do not possess any tracheal system (Xué *et al.*, 1993), oxygen uptake via the cuticle is probably sufficient because of their tiny size and low motility (Pass and Szucsich, 2011); thus, the haemocyanins may have been lost in this group. Diplura have developed trachea with longitudinal connections between the tracheae from spiracles, and the respiratory protein therefore appears to be dispensable, owing to the poor mobility of these species (Paclt, 1956). In Collembola, most species do not possess any tracheal system (Davies, 1927), and haemocyanin is necessary to transport oxygen, especially in certain energetic lineages with strong furca, eg order Entomobryomorpha. Flachsbarth *et al.* (2017) investigated the distribution of haemocyanin in Collembola, and found Hc2 was widespread amongst various collembolan lineages, whereas Hc1 was only detected in Entomobryomorpha.

Hexamerins have been demonstrated to be indispensable in many insect orders, playing roles as storage proteins (Telfer and Kunkel, 1991; Beintema *et al.*, 1994; Burmester *et al.*, 1998; Sánchez *et al.*, 1998; Burmester, 1999; Wheeler *et al.*, 2000; Zhang *et al.*, 2015). Hexamerins were also recently identified in Diplura, a group of basal hexapods (Pick and Burmester, 2009; Xie and Luan, 2014). In this study, we verified the occurrence of hexamerins and cloned two complete sequences from one of the most primitive hexapod lineages, Collembola (Table 1), and we found they are exclusively expressed in the embryonic stage (Fig. 2), unlike most insect hexamerins, which are highly expressed during post‐embryonic development (Burmester, 2015b). Collembolans do not undergo metamorphosis but moult every three days for their entire lifespan (Cutkomp *et al.*, 1987), and the moulting process is rapid. Thus, it is possible that hexamerin is not necessary to survive the short period of starvation during moulting. The embryonic stage is the only long nonfeeding stage during the life cycle of collembolans, and collembolan hexamerins may support the development process by serving as a nutrition pool. Our developmental transcriptome analysis suggests that the collembolan hexamerins are related to the JH biosynthesis, riboflavin metabolism and energy or nutrition (eg amino acids, sucrose, folate) metabolic pathways (Dataset S1 and Table S1). These functions are very similar to those have been reported in insect hexamerins (Pan and Telfer, 1999; Wheeler *et al.*, 2000). Accordingly, collembolan hexamerins probably serve as storage proteins and exhibit similar functions to other hexapod hexamerins, which is also supported by the strong purifying selection upon hexapod hexamerins.

In Protura, we identified three possible hexamerin sequences (61−81 amino acids) in the transcriptome of *Acerentomon* sp., but these sequences were too short for validation and phylogenetic analysis (data not shown). We attempted but failed to acquire hexamerin sequences via PCR using cDNA from proturan juveniles and adults. It is possible that proturan hexamerins are also solely expressed in the embryonic period. However, in Diplura, which is currently recognized as the closest relative of Insecta, hexamerins were amplified from the cDNA of dipluran juveniles and adults. Regrettably, the inaccessibility of proturan and dipluran eggs prevented us from studying embryonic expression in these groups.

### Origins and evolution of hexamerins in Hexapoda

In our phylogenetic tree (Fig. 3), the collembolan hexamerins joined the clade of dipluran hexamerins, and they are a sister group to the clade of insect hexamerins. Pick and Burmester (2009) reported the first dipluran hexamerin and proposed that hexamerins evolved independently at least two times in Hexapoda; however, Xie and Luan (2014) supported a single origin of the hexamerins in Hexapoda after adding eight dipluran hexamerin sequences into the phylogenetic analyses. In the present study, the discovery of collembolan hexamerins and corresponding phylogenetic analysis constitute new strong evidence for the monophyletic origin of hexapod hexamerins. Notably, all three collembolan hexamerins displayed remarkably long branches in our study (Fig. 3), suggesting they may have accumulated more mutations than other hexamerins.

The ancient origins of hexamerins are now uncovered, and the possible driving forces of this critical evolutionary scenario are to be explored. As indicated by the molecular dating results, hexamerins originated from haemocyanins approximately 423 Ma in the late Silurian–early Devonian period, concurrent with the increase of atmospheric oxygen levels (Fig. 4; Berner, 2009; Schachat *et al.*, 2018). More oxygen could freely flow through the cuticle and even the original trachea, and the respiratory proteins may have become less important in Collembola to some extent, which may have allowed some haemocyanins to evolve. In fact, Hc1 has been lost in some collembolan lineages (Flachsbarth *et al.*, 2017).

## Experimental procedures

### Animal collections

The two strains of the collembolan *F. candida* used in this work (the DK strain from Amsterdam, the Netherlands, and the SH stain from Shanghai, China) have been bred in our laboratory for more than 10 years. These collembolans are usually fed with baker’s yeast and reared in Petri dishes with mixed solidified plaster of paris and activated charcoal (9:1 weight, dissolved in distilled water, approximately 270 ml water/500 g of the powdered mixture) covering the bottom. The life histories of parthenogenetic *F. candida* (DK) and bisexual *F. candida* (SH) are very similar. At 21 ℃, the eggs take 7 to 10 days to hatch, and three additional weeks are required to reach sexual maturity (Fountain and Hopkin, 2005).

The living diplurans *L. weberi* and *Occ. japonicus* were collected in Shanghai, whereas the dipluran *Oct. sinensis* was collected from Zhanjiang, Guangdong Province, China.

### Sequence acquisitions

To clone hexamerin and haemocyanin sequences via PCR amplification, total RNA was extracted from pooled specimens of living eggs, juveniles and adults of the collembolans *F. candida* (DK) and *F. candida* (SH) using QIAzol Lysis Reagent (QIAGEN, Valencia, CA, USA), whereas the RNA of three dipluran species was prepared from living adult specimens. First‐strand cDNA synthesis was performed with SuperScript III Reverse Transcriptase (Invitrogen, Carlsbad, CA, USA) according to the manufacturer’s instructions. Partial sequences of the collembolan and dipluran hexamerins were obtained via reverse‐transcript PCR using degenerate primers, and the complete sequences were obtained with a SMARTer RACE 5′/3′ kit (Takara Bio Company, Mountain View, CA, USA). All primers used in these assays are listed in Table S4.

In addition, we screened haemocyanin and hexamerin sequences from the transcriptomes of basal hexapods sequenced by Misof *et al. *(2014), Faddeeva *et al. *(2015) and in our laboratory (unpublished data), including six collembolans, four diplurans and two proturans. Hexamerin genes from the zygentoman *Thermobia domestica* (*TdoHx*; Pick *et al.*, 2008) and the dipluran *Campodea* sp. (*CspHx1*; Pick and Burmester, 2009) and *L. weberi* (*LweHx1*; Xie and Luan, 2014) and haemocyanin genes from the zygentoman *T. domestica* (*TdoHc1* and *TdoHc2*; Pick *et al.*, 2008) were used as query sequences. The tblastn program (version 2.7.1) was run locally against these transcriptome databases using the blast software (Altschul *et al.*, 1990) downloaded from the NCBI website and Sequenceserver (1.0.11) (Priyam *et al.*, 2015), with a threshold E‐value of 1e‐5. The hits were sorted and checked by PCR amplification, then sequenced and used in phylogenetic analyses for putative haemocyanin family members.

### Semi‐quantitative RT‐PCR

First, *F. candida* (DK) was synchronized for three generations: some adult individuals (F_0_ generation) were transferred to a new Petri dish for 24 h of oviposition, and the eggs (F_1_ generation) were subsequently transferred to a new Petri dish for 1 month of culture, to reach sexual maturity. The procedure was repeated twice to obtain adults of the F_3_ generation.

Approximately 300 synchronized adults of the F_3_ generation were then transferred to three new Petri dishes for 24 h of oviposition, and all eggs were transferred to a new culture dish. Next, all eggs laid by the F_3 _generation were collected and transferred to new culture dishes every 24 h. After 31 days of collection and culture, we obtained collembolans covering all developmental stages from the first to the 31st day (including eggs, juveniles and adults). On the 31st day, RNA was prepared individually from 31 samples using a miRNeasy mini kit (QIAGEN) according to the manual.

First‐strand cDNA was synthesized using a TransScript One‐Step gDNA Removal and cDNA Synthesis SuperMix kit (Beijing TransGen Biotech Co., Ltd, Beijing, China) according to the manual. *β‐tubulin* of *F. candida* (DK) was used as the control gene for RT‐PCR. In the analysis of expression profiles, we normalized the cDNA samples by amplifying the *β‐tubulin* gene for 28 cycles, whereas hexamerin *Fca(DK)Hx1* and haemocyanin *Fca(DK)Hc2* were both amplified for 35 cycles. All primers used in these assays are listed in Table S4.

### RNA sequencing and mining of correlated genes

Following the synchronization procedure described above, 15 developmental samples of *F. candida* (DK), including eggs (each day from days 0.5 to 9.5), juveniles (days 12.5, 19.5 and 28.5) and adults (days 31.5 and 45.5), were chosen for transcriptome sequencing. RNA was extracted as indicated above, and transcriptomes were sequenced on the BGISEQ‐500 platform (Goodwin *et al.*, 2016) by BGI (Shenzhen, China).

The combined genomic data for *F. candida* (DK) from Faddeeva‐Vakhrusheva *et al*. (2017) and our laboratory (unpublished data) were used as the reference for the transcriptome analyses. The genome index was built using bowtie2 (Langmead and Salzberg, 2012), and the reads were mapped using tophat2 (Kim *et al.*, 2013). The transcript abundance (RPKM) of *Fca(DK)Hx1* and *Fca(DK)Hc2* was estimated using cufflinks (Trapnell *et al.*, 2012).

To infer the putative function of collembolan hexamerins, we used the Spearman correlation method (*r* ^2 ^≥ 0.9, *P* < 0.05) to explore the genes correlated with *Fca(DK)Hx1* during embryonic development with the transcriptomes sequenced from 10 embryonic stages. The correlated transcripts were sorted, and gene ontology and KEGG pathways were annotated and classified using blast2go (Kanehisa and Goto, 2000; Conesa *et al.*, 2005).

### Selective pressure analyses

10 Hc1, 10 Hc2 and 40 Hx were used for the selective pressure analysis, including 51 full‐length cDNA sequences of hexapod haemocyanins and hexamerins downloaded from NCBI and nine new complete cDNA sequences [*PloHc1*, *OciHc2*, *PloHc2*, *LweHx2*, *LweHx3*, *OsiHx1*, *OsiHx3*, *Fca(DK)Hx1* and *Fca(SH)Hx1*], covering Collembola, Diplura, Zygentoma, Plecoptera, Blattodea, Orthoptera, Neoptera, Hymenoptera, Coleoptera, Lepidoptera and Diptera (details of all sequences are shown in Table S5). Each dataset was aligned by codon with prank (Loytynoja, 2014), with the stop codons and unaligned sequences deleted manually (Datasets S4, S5 and S6). Maximum likelihood trees based on three aligned datasets were reconstructed with mega7 (Kumar *et al.*, 2016), respectively, and were used as the guide trees in the analyses (Figs S4, S5 and S6). By using the codeml package in paml (version 4.9c) (Yang, 2007), we performed the M0 model (one ratio) to estimate the selective pressures on hexapod Hc1, Hc2 and Hx, respectively, with the α parameter fixed at 0 (model of a single rate for all sites), and other parameters as default values.

### Phylogenetic analysis and molecular clock estimation

A total of 130 pancrustacean haemocyanin, cryptocyanin and hexamerin sequences were used for phylogenetic reconstruction, including 113 genes reported previously (Table S3) and 17 new genes (Table 1). A multiple sequence alignment was performed with mafft (http://mafft.cbrc.jp/alignment/server/; Katoh *et al.*, 2017) using the L‐INS‐I method, and the result is presented in Dataset S3, as visualized using genedoc v. 2.7 (Nicholas *et al.*, 1997). The appropriate model for WAG + Gamma was selected with prottest (Abascal *et al.*, 2005) using the Akaike information criterion. We performed Bayesian analysis with mrbayes 3.2.6 (Huelsenbeck and Ronquist, 2001). The prior probabilities were equal for all trees. Metropolis‐coupled Markov chain Monte Carlo sampling was run for 4 000 000 generations with one cold chain and three heated chains. The average split frequencies were stationary and below 0.01 after approximately 1 600 000 generations. The starting trees were randomly selected. Trees were sampled every 100 generations, and the burn‐in value was set to 20 000 to estimate the posterior probabilities. Crustacean haemocyanins and cryptocyanins were set as the outgroup. raxml v. 8.2.10 (Stamatakis, 2006) was applied for maximum likelihood analysis, with the bootstrap value set to 1000. All phylogenetic analyses were run on Cyberinfrastructure for Phylogenetic Research (CIPRES) Science Gateway v. 3.3 (http://www.phylo.org/; Miller *et al*., 2010).

We employed the phylogenetic tree reconstructed through raxml to estimate the divergence time of the hexapod hexamerins. The molecular clock analysis was performed using the RelTime method (Tamura *et al.*, 2012) with mega7 (Kumar *et al.*, 2016). The earliest collembolan fossil (*Rhyniella praecursor*, 411.6 Ma; Whalley and Jarzembowski, 1981; Garrouste *et al.*, 2012) was used for calibration. The assumed divergence times for Diptera and Lepidoptera (between 281.5 and 307.2 Ma) and for Brachycera and Nematocera (between 238.5 and 295.4 Ma) (Hagner‐Holler *et al.*, 2007; Pick *et al.*, 2008) were also applied for calibration. All crustacean haemocyanins and cryptocyanins were set as the outgroup. In addition, the palaeontological oxygen concentration curve (Berner, 2009) was mapped onto the time‐estimated tree to deduce the relationship between environmental conditions and hexamerin emergence.

## Supporting information


**Figure S1.** Gene ontology profiles of genes co expressed with *Folsomia candida (Denmark strain) hexamerin 1*.

**Figure S2.** Gene ontology profiles of genes co expressed with* Folsomia candida (Denmark strain) haemocyanin 2*.

**Figure S3.** Original tree for Fig. 3.

**Figure S4.** Guidance tree of type 1 haemocyanins used for selective pressure analysis.

**Figure S5.** Guidance tree of type 2 haemocyanins used for selective pressure analysis.

**Figure S6.** Guidance tree of hexamerins used for selective pressure analysis.

**Table S1.** Kyoto encyclopedia of genes and genomes (KEGG) pathways of genes co‐expressed with *Folsomia candida (Denmark strain) hexamerin 1*.

**Table S2.** Kyoto encyclopedia of genes and genomes (KEGG) pathways of genes co‐expressed with* Folsomia candida (Denmark strain) haemocyanin 2*.

**Table S3.** List of 113 previously reported sequences used in the phylogenetic construction.

**Table S4.** List of all primers employed in this study.

**Table S5.** List of 60 nucleotide sequences used in selective pressure analyses.

**Dataset S1.** The 164 transcripts correlated with *Folsomia candida (Denmark strain) hexamerin 1 *during embryonic stages.

**Dataset S2.** The 1069 genes correlated with* Folsomia candida (Denmark strain) haemocyanin 2 *during embryonic stages.

**Dataset S3.** Multiple sequence alignment of 130 amino acid sequences employed in the phylogenetic construction.

**Dataset S4.** Ten type 1 haemocyanins employed in the selective pressure analysis.

**Dataset S5.** Ten type 2 haemocyanins employed in the selective pressure analysis.

**Dataset S6.** Forty hexamerins employed in the selective pressure analysis.
