# Supplementary figures and images for: Developmental expression and evolution of hexamerin and haemocyanin from Folsomia candida (Collembola)
Source: Insect Mol Biol. 2019 May 8;28(5):716–27. doi: 10.1111/imb.12585 (PMC6850205; doi:10.1111/imb.12585)

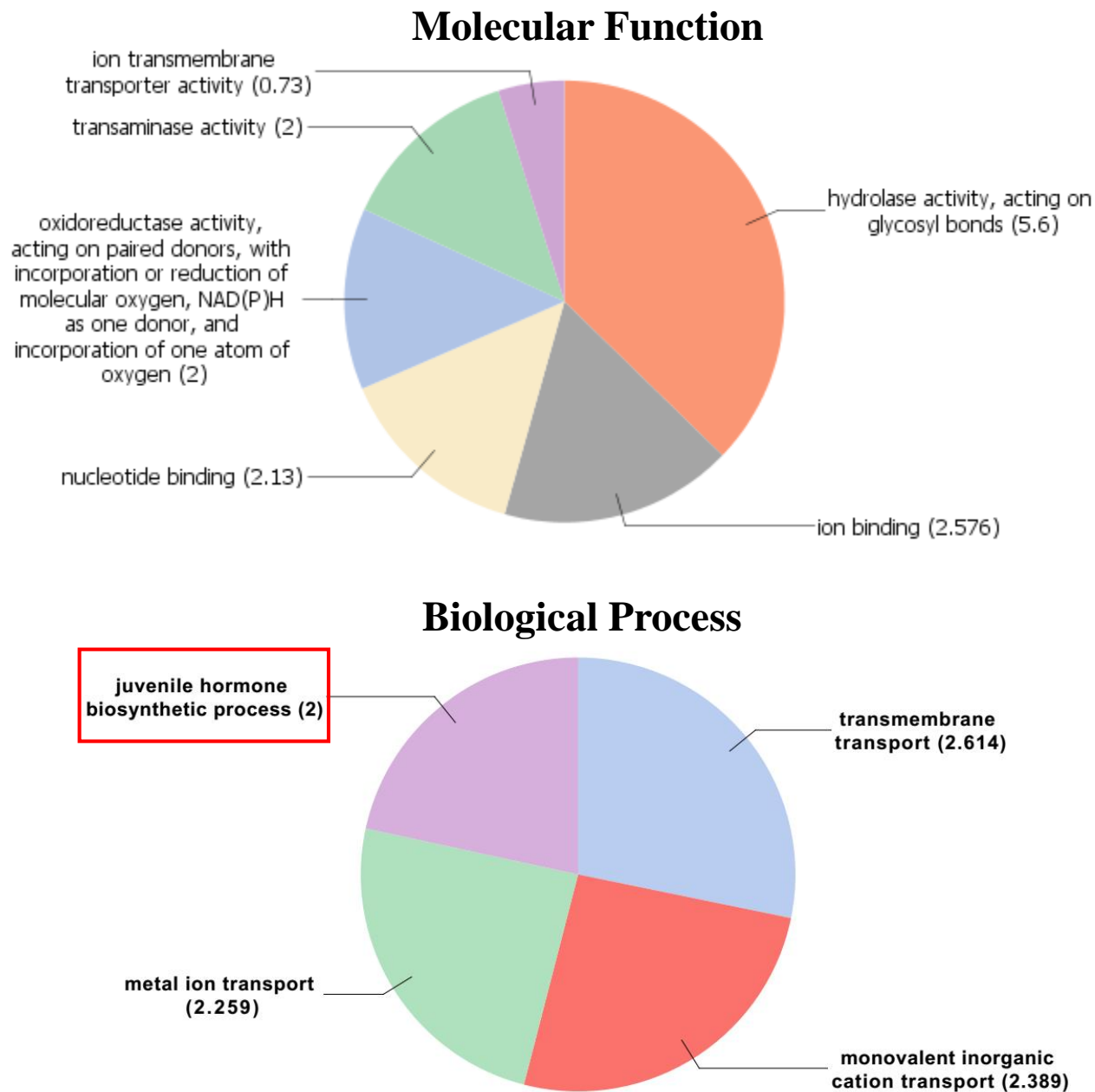

Figure S1. GO profiles of genes co-expressed with *Fca(DK)Hx1*.

Supplement: Supplementary file 1 — Figure S1. Gene ontology profiles of genes co expressed with Folsomia candida (Denmark strain) hexamerin 1. [file IMB-28-716-s001.pdf]
