# Supplementary material for: Developmental expression and evolution of hexamerin and haemocyanin from Folsomia candida (Collembola)
Source: Insect Mol Biol. 2019 May 8;28(5):716–27. doi: 10.1111/imb.12585 (PMC6850205; doi:10.1111/imb.12585)
Supplement: Supplementary file 2 — Figure S2. Gene ontology profiles of genes co expressed with Folsomia candida (Denmark strain) haemocyanin 2. [file IMB-28-716-s002.pdf]

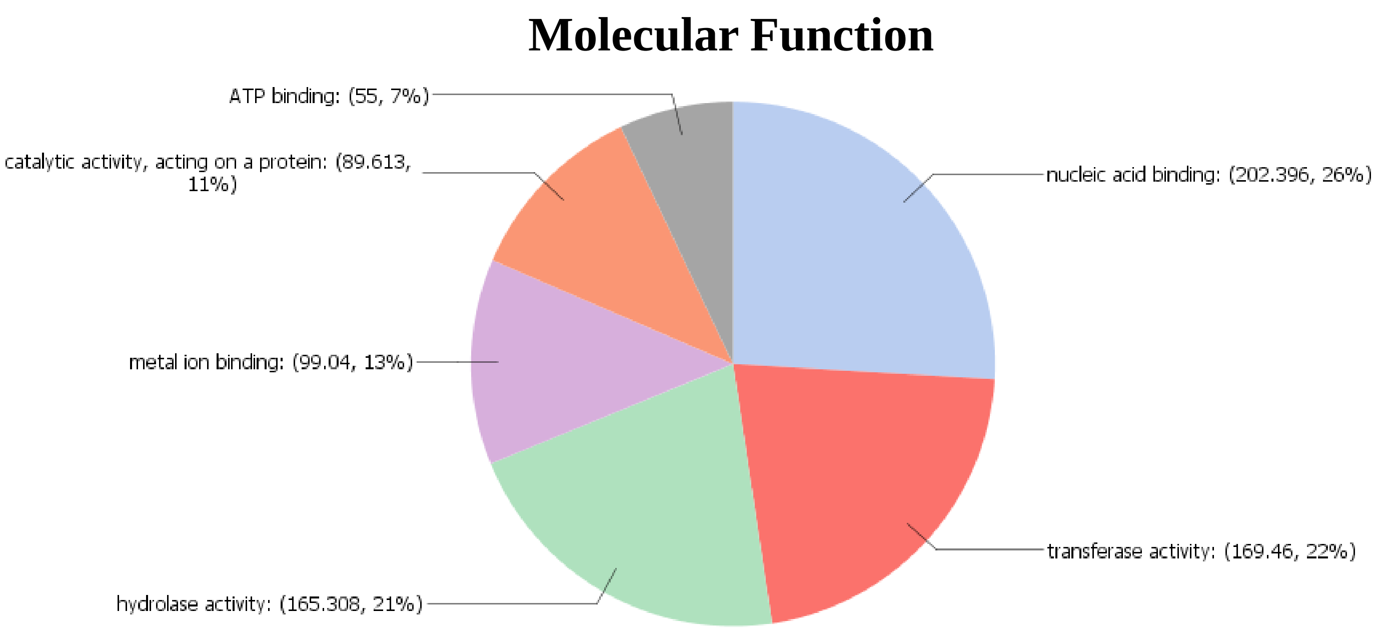

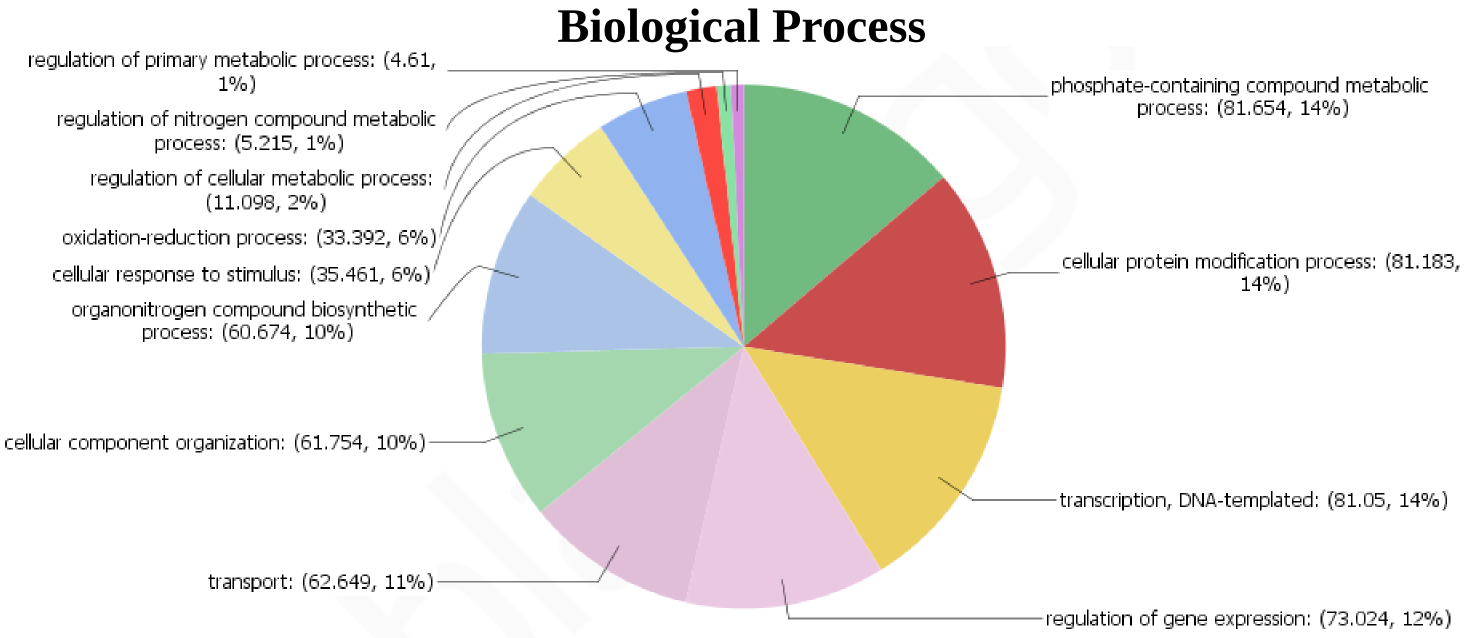

Figure S2. GO profiles of genes co-expressed with *Fca(DK)Hc2*.
