# Supplementary material for: Developmental expression and evolution of hexamerin and haemocyanin from Folsomia candida (Collembola)
Source: Insect Mol Biol. 2019 May 8;28(5):716–27. doi: 10.1111/imb.12585 (PMC6850205; doi:10.1111/imb.12585)
Supplement: Supplementary file 3 — Figure S3. Original tree for Fig. 3. [file IMB-28-716-s003.pdf]

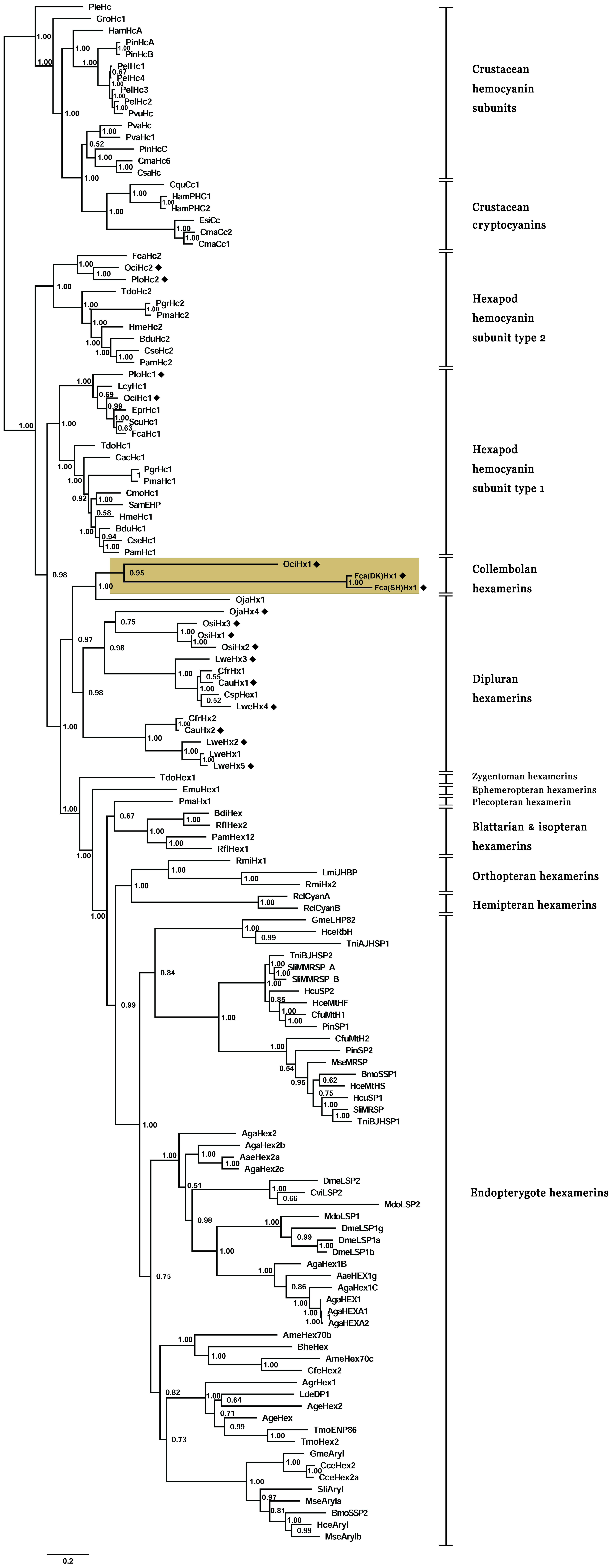

Figure S3. Original tree for Fig. 3. Numbers at the nodes indicate the Bayesian posterior probabilities. New sequences added in this study are marked with black squares. The full species names and protein names for all abbreviations are listed in Dataset S3 and Table 1.
