# Supplementary material for: Developmental expression and evolution of hexamerin and haemocyanin from Folsomia candida (Collembola)
Source: Insect Mol Biol. 2019 May 8;28(5):716–27. doi: 10.1111/imb.12585 (PMC6850205; doi:10.1111/imb.12585)
Supplement: Supplementary file 5 — Figure S5. Guidance tree of type 2 haemocyanins used for selective pressure analysis. [file IMB-28-716-s005.pdf]

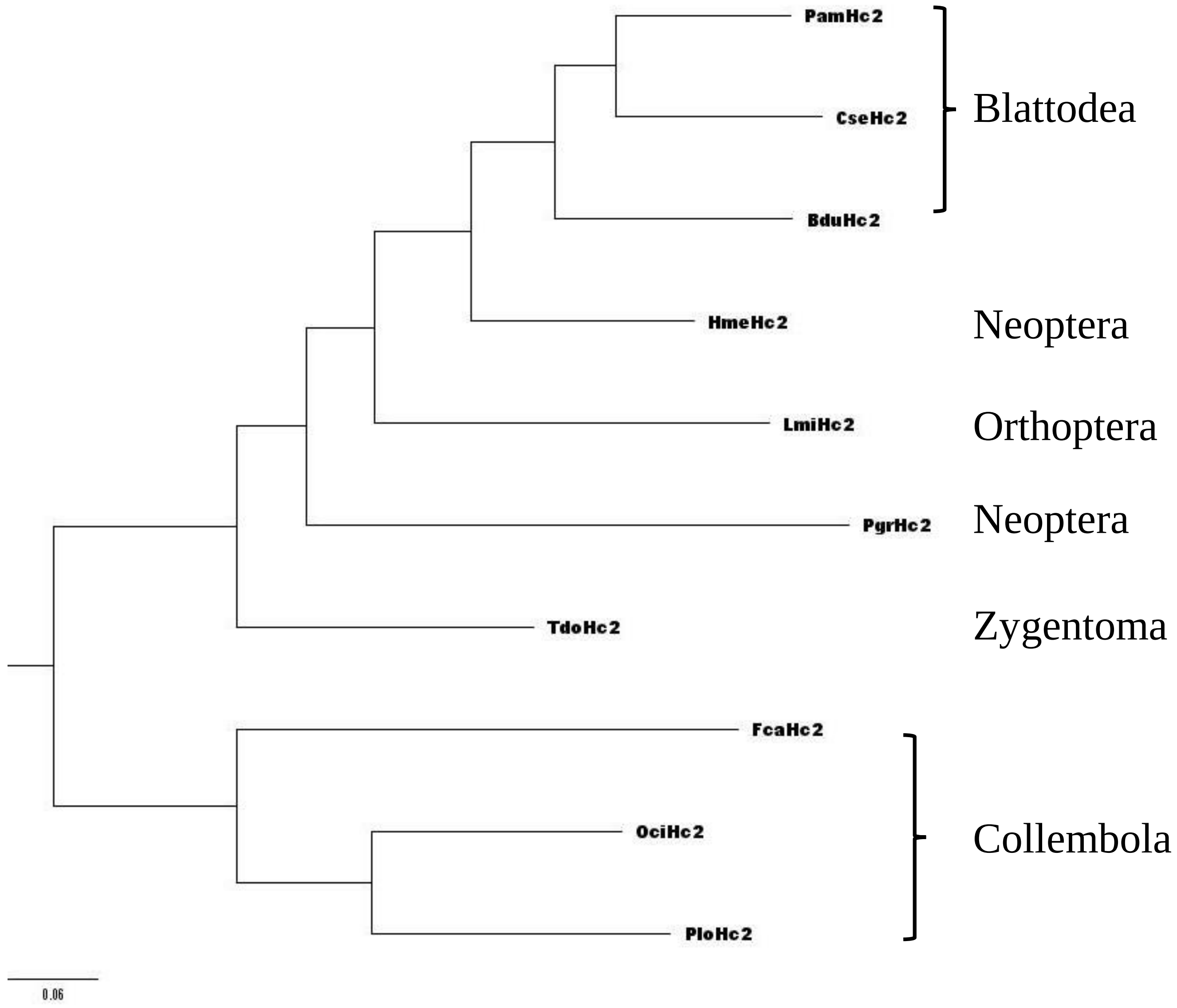

Figure S5. Guidance tree of hexapod Hc2 used to conduct selective pressure analysis in PAML. Abbreviations of genes are listed Table S5.
