# Supplementary material for: Developmental expression and evolution of hexamerin and haemocyanin from Folsomia candida (Collembola)
Source: Insect Mol Biol. 2019 May 8;28(5):716–27. doi: 10.1111/imb.12585 (PMC6850205; doi:10.1111/imb.12585)
Supplement: Supplementary file 9 — Table S3. List of 113 previously reported sequences used in the phylogenetic construction. [file IMB-28-716-s009.docx]

**Table S3. List of 113 previously reported sequence information used in the phylogenetic construction.**

| **Molecule Abbreviation** | **GenBank Accession No.** | **Protein** | **Species** | **Groups** |
| --- | --- | --- | --- | --- |
| HamHcA | AJ272095 | hemocyanin SU A | *Homarus americanus* | Crustacea |
| PinHcB | P10787 | hemocyanin SU b | *Panulirus interruptus* | Crustacea |
| PinHcA | P04254 | hemocyanin SU a | *Panulirus interruptus* | Crustacea |
| PinHcC | S21221 | hemocyanin C | *Panulirus interruptus* | Crustacea |
| PvaHc1 | AJ250830 | hemocyanin SU 1 | *Penaeus vannamei* | Crustacea |
| PvaHc | X82502 | hemocyanin | *Penaeus vannamei* | Crustacea |
| PelHc1 | AJ344361 | hemocyanin SU 1 | *Palinurus elephas* | Crustacea |
| PelHc2 | CAC69244 | hemocyanin SU 2 | *Palinurus elephas* | Crustacea |
| PelHc3 | CAC69245 | hemocyanin SU 3 | *Palinurus elephas* | Crustacea |
| PelHc4 | CAD56697 | hemocyanin SU 4 | *Palinurus elephas* | Crustacea |
| PvuHc | P80888 | hemocyanin | *Palinurus vulgaris* | Crustacea |
| GroHc1 | CAI78901 | hemocyanin SU 1 | *Gammarus roeseli* | Crustacea |
| PleHc | AF522504 | hemocyanin | *Pacifastacus leniusculus* | Crustacea |
| CsaHc | AF249297 | hemocyanin SU | *Callinectes sapidus* | Crustacea |
| CmaHc6 | U48881 | hemocyanin SU 6 | *Cancer magister* | Crustacea |
| EsiCc1 | AGH32536.1 | cryptocyanin 1 | *Eriocheir sinensis* | Crustacea |
| CquCc1 | ALC79582.1 | cryptocyanin 1 | *Cherax quadricarinatus* | Crustacea |
| CmaCc1* | AAD09762.1,  MG952979 | cryptocyanin 1 | *Cancer magister* | Crustacea |
| CmaCc2 | ABB59714.1 | cryptocyanin 2 | *Cancer magister* | Crustacea |
| HamPHc1 | AJ132141 | pseudohemocyanin 1 | *Homarus americanus* | Crustacea |
| HamPHc2 | Q6KF81 | pseudohemocyanin 2 | *Homarus americanus* | Crustacea |
| ScuHc1 | FM242638 | hemocyanin SU 1 | *Sinella curviseta* | Collembola |
| FcaHc1 | FM242639 | hemocyanin SU 1 | *Folsomia candida* | Collembola |
| EprHc1 | AGR40410 | hemocyanin SU 1 | *Entomobrya proxima* | Collembola |
| LcyHc1 | AGR40409 | hemocyanin SU 1 | *Lepidocyrtus cyaneus* | Collembola |
| CspHex1 | CAX63173 | hexamerin 1 | *Campodea* sp. CP-2009 | Diplura |
| OjaHx1 | AGR40405 | hexamerin 1 | *Occasjapyx japonicus* | Diplura |
| LweHx1 | AGR40404 | hexamerin 1 | *Lepidocampa weberi* | Diplura |
| CfrHx1 | AGR40411 | hexamerin 1 | *Campodea fragilis* | Diplura |
| CfrHx2 | AGR40412 | hexamerin 2 | *Campodea fragilis* | Diplura |
| PgrHc1 | DQ118369 | hemocyanin SU 1 | *Perla grandis* | Insecta |
| PmaHc1 | AJ555403 | hemocyanin SU 1 | *Perla marginata* | Insecta |
| TdoHc1 | FM165288 | hemocyanin SU 1 | *Thermobia domestica* | Insecta |
| CacHc1 | FM242641 | hemocyanin SU 1 | *Chelidurella acanthopygia* | Insecta |
| CmoHc1 | FM242640 | hemocyanin SU 1 | *Carausius morosus* | Insecta |
| HmeHc1 | FM242642 | hemocyanin SU 1 | *Hierodula membranacea* | Insecta |
| BduHc1 | FM242646 | hemocyanin SU 1 | *Blaptica dubia* | Insecta |
| CseHc1 | FM242644 | hemocyanin SU 1 | *Cryptotermes secundus* | Insecta |
| PamHc1 | FM242648 | hemocyanin SU 1 | *Periplaneta americana* | Insecta |
| SamEHP | AF038569 | embryonic hemolymph protein | *Schistocerca americana* | Insecta |
| FcaHc2 | KF670722 | hemocyanin SU 2 | *Folsomia candida* | Insecta |
| PgrHc2 | DQ118370 | hemocyanin SU 2 | *Perla grandis* | Insecta |
| PmaHc2 | AJ555404 | hemocyanin SU 2 | *Perla marginata* | Insecta |
| TdoHc2 | FM165289 | hemocyanin SU 2 | *Thermobia domestica* | Insecta |
| HmeHc2 | FM242643 | hemocyanin SU 2 | *Hierodula membranacea* | Insecta |
| BduHc2 | FM242647 | hemocyanin SU 2 | *Blaptica dubia* | Insecta |
| CseHc2 | FM242645 | hemocyanin SU 2 | *Cryptotermes secundus* | Insecta |
| PamHc2 | FM242649 | hemocyanin SU 2 | *Periplaneta americana* | Insecta |
| TdoHex1 | FM165290 | hexamerin 1 | *Thermobia domestica* | Insecta |
| EmuHex1 | Unpublished | hexamerin 1 | *Ephemerella mucronata* | Insecta |
| PmaHex1 | AM690365 | hexamerin 1 | *Perla marginata* | Insecta |
| RflHex1 | AY572858 | hexamerin 1 | *Reticulitermes flavipes* | Insecta |
| PamHex12 | L40818 | hexamerin (allergen clone C12) | *Periplaneta americana* | Insecta |
| BdiHex | U31328 | hexamerin | *Blaberus discoidalis* | Insecta |
| RflHex2 | AY572859 | hexamerin 2 | *Reticulitermes flavipes* | Insecta |
| RmiHx1 | AY923852 | hexamerin 1 | *Romalea microptera* | Insecta |
| LmiJHBP | U74469 | juvenile hormone binding protein | *Locusta migratoria* | Insecta |
| RmiHx2 | AY923851 | hexamerin 2 | *Romalea microptera* | Insecta |
| RclCyanA | D87272 | cyanoprotein alpha subunit | *Riptortus clavatus* | Insecta |
| RclCyanB | D87273 | cyanoprotein beta subunit | *Riptortus clavatus* | Insecta |
| GmeLHP82 | L21997 | LHP82 | *Galleria mellonella* | Insecta |
| HceRbH | AF032397 | riboflavin binding hexamerin | *Hyalophora cecropia* | Insecta |
| TniAJHSP1 | M57443 | acidic juvenile hormone-suppressible protein | *Trichoplusia ni* | Insecta |
| HceMtHF | AF032398 | moderately methionine-rich hexamerin | *Hyalophora cecropia* | Insecta |
| PinSP1 | AF356842 | hexamerin storage protein P1 | *Plodia interpunctella* | Insecta |
| CfuMtH1 | AF007767 | diapause associated protein | *Choristoneura fumiferana* | Insecta |
| HcuSP2 | AF157013 | storage protein-2 | *Hyphantria cunea* | Insecta |
| TniBJHSP2 | L03281 | basic juvenile hormone-suppressible protein 2 | *Trichoplusia ni* | Insecta |
| SliMMRSP_B | AJ249468 | moderately methionine-rich storage protein α | *Spodoptera litura* | Insecta |
| SliMMRSP_A | AJ249469 | moderately methionine-rich storage protein β | *Spodoptera litura* | Insecta |
| CfuMtH2 | AF007768 | diapause associated protein | *Choristoneura fumiferana* | Insecta |
| PinSP2 | AF356843 | hexamerin storage protein P2 | *Plodia interpunctella* | Insecta |
| HcuSP1 | U60988 | storage protein-1 | *Hyphantria cunea* | Insecta |
| TniBJHSP1 | L03280 | basic juvenile hormone-suppressible protein 1 | *Trichoplusia ni* | Insecta |
| SliMRSP | AJ249470 | methionine rich-storage protein | *Spodoptera litura* | Insecta |
| MseMRSP | L07609 | methionine rich-storage protein | *Manduca sexta* | Insecta |
| BmoSSP1 | X12978 | Sex-specific storage protein 1 | *Bombyx mori* | Insecta |
| HceMtHS | AF032399 | very methionine rich storage protein | *Hyalophora cecropia* | Insecta |
| GmeAryl | M73793 | arylphorin | *Galleria mellonella* | Insecta |
| CceHex2a | AF294808 | hexamerin 2a | *Corcyra cephalonica* | Insecta |
| CceHex2 | AF294809 | hexamerin 2b | *Corcyra cephalonica* | Insecta |
| MseAryla | M28396 | arylphorin alpha | *Manduca sexta* | Insecta |
| SliAryl | AJ249471 | arylphorin | *Spodoptera litura* | Insecta |
| BmoSSP2 | P20613 | sex-specific storage protein-2 (arylphorin) | *Bombyx mori* | Insecta |
| HceAryl | AF032396 | arylphorin | *Hyalophora cecropia* | Insecta |
| MseArylb | M28397 | arylphorin beta | *Manduca sexta* | Insecta |
| AgaHex2 | XM_321800 | hexamerin 2 | *Anopheles gambiae* | Insecta |
| AgaHex2b | XM_551147 | hexamerin 2 beta | *Anopheles gambiae* | Insecta |
| AaeHex2a | U86080 | hexamerin 2 alpha | *Aedes aegypti* | Insecta |
| AgaHex2c | XM_551149 | hexamerin 2c | *Anopheles gambiae* | Insecta |
| DmeLSP2 | AC023678 | LSP-2 | *Drosophila melanogaster* | Insecta |
| MdoLSP2**^$^** | Capurro et al., 1997**^$^** | LSP-2 | *Musca domestica* | Insecta |
| CviLSP2 | U89789 | LSP-2 | *Calliphora vicina* | Insecta |
| MdoLSP1 | AY256680 | hexamerin L1 | *Musca domestica* | Insecta |
| DmeLSP1g | NM_079144 | LSP-1 gamma | *Drosophila melanogaster* | Insecta |
| DmeLSP1b | NM_057276 | LSP-1 beta | *Drosophila melanogaster* | Insecta |
| DmeLSP1a | NM_078583 | LSP-1 alpha | *Drosophila melanogaster* | Insecta |
| AgaHex1B | XM_311376 | hexamerin 1B | *Anopheles gambiae* | Insecta |
| AaeHEX1g | U86079 | hexamerin 1 gamma | *Aedes aegypti* | Insecta |
| AgaHex1C | AAAB01008960 | hexamerin 1C | *Anopheles gambiae* | Insecta |
| AgaHEX1 | AF020870 | hexamerin 1 | *Anopheles gambiae* | Insecta |
| AgaHEXA1 | U51225 | hexamerin A1 | *Anopheles gambiae* | Insecta |
| AgaHEXA2 | AF020871 | hexamerin A2 | *Anopheles gambiae* | Insecta |
| AmeHex70b | AY601637 | hexamerin 70b | *Apis mellifera* | Insecta |
| BheHex | I25974 | Insecticidal toxin (hexamerin) | *Bracon hebetor* | Insecta |
| AmeHex70c | XM_392869 | hexamerin 70c | *Apis mellifera* | Insecta |
| CfeHex2 | AJ251271 | hexamerin 2 | *Camponotus festinatus* | Insecta |
| LdeDP1 | X76080 | diapause protein 1 | *Leptinotarsa decemlineata* | Insecta |
| AgrHex1 | AF512519 | SP-1 arylphorin | *Anthonomus grandis* | Insecta |
| AgeHex2 | AY293287 | arylphorin-like hexamerin 2 | *Apriona germari* | Insecta |
| AgeHex | AF509880 | arylphorin-like hexamerin 1 | *Apriona germari* | Insecta |
| TmoHex2 | AF395329 | hexamerin 2 | *Tenebrio molitor* | Insecta |
| TmoENP86 | AB021700 | Enp-6 (86 kDa early-stage hexamerin) | *Tenebrio molitor* | Insecta |

***** CmaCc1 from *Cancer magiste**r* reported previously (Terwilliger et al., 1999) was extended to the start codon at the N-terminal considering the relevant contigs (Phillips et al., unpublished: FG310395.1, FG310383.1, FG309576.1, FG310212.1, FG310455.1, FG310202.1), the assession number of the sequence we employed in this study is MG952979.

**^$^** MdoLSP2 from *Musca domesticais* was reported by Capurro et al. (1997).

References

Capurro, M.D., Marinotti, O. , Farah, C.S., James, A.A., Bianchi, A.G. (1997) The nonvitellogenic female protein of *Musca domestica* is an adult-specific hexamerin. *Insect Mol Bio* **6**: 97-104.

Terwilliger, N.B., Dangott, L., Ryan, M. (1999) Cryptocyanin, a crustacean molting protein: evolutionary link with arthropod hemocyanins and insect hexamerins. *Proc Natl Acad Sci* **96**: 2013-2018.
